# Characteristics of Force Development and Muscle Excitation in Resisted and Assisted Jumps in Comparison with the Isometric Mid-Shin Pull

**DOI:** 10.3390/s25030975

**Published:** 2025-02-06

**Authors:** Giuseppe Rosaci, Davide Latini, Federico Nigro, Sandro Bartolomei

**Affiliations:** Department for Life Quality Studies, University of Bologna, 40126 Bologna, Italy; giuseppe.rosaci@unibo.it (G.R.); davide.latini22@gmail.com (D.L.); sandro.bartolomei@unibo.it (S.B.)

**Keywords:** peak rate of force development, power, electromyography, resistance band, assisted band

## Abstract

Background: The purpose of this study was to examine the relationships between the characteristics of force development and electromyographic activity of the quadriceps muscles in the isometric mid-shin pull (MSP) and the countermovement jump (CMJ) performed under different conditions. Methods: Fifteen resistance-trained individuals (age = 25.9 ± 4.0 y; body mass = 73.2 ± 11.7 Kg; stature = 172.3 ± 9.5 cm) were tested for MSP and for the following CMJs: regular CMJ (CMJ); elastic band-assisted CMJ (CMJ_AB_); elastic band-resisted CMJ (CMJ_RB_); weighted vest CMJ (CMJ_V_) in random order, using a force plate. Peak force (PF) and peak rate of force development (PRFD) were calculated in all the assessments, while peak velocity and power were calculated only in the CMJs. In addition, during all the tests, electromyographic activity of the vastus lateralis (EMG_VL_) and of vastus medialis (EMG_VM_) was detected. Results: Higher PF was registered in MSP compared to the CMJs (*p* < 0.001). PRFD and EMG_VL_ were significantly more elevated in the CMJs compared to the MSP (*p* < 0.05). No significant correlations were noted between the PRFD measured in MSP and in CMJs, while the PRFD in MSP was largely correlated with PP in CMJs (r = 0.68/0.83). Conclusions: Results of the present study showed that CMJs promote PRFD and the excitation of the vastus lateralis, to a greater extent compared to MSP. Regular CMJ performed at body mass may represent the best option for power development, and small variations in loads allowed by weighted vests or elastic bands do not seem to alter the characteristics of force development.

## 1. Introduction

Explosive strength and power represent key factors for success in several sport disciplines and many resistance training programs are focused on these components [1,2]. The rate of force development (RFD) is a widely recognized parameter of explosive strength that has been used to assess the force–time characteristics of isometric muscle actions [3,4]. The RFD and the peak rate of force development (PRFD) have also been determined in dynamic muscle actions, such as weightlifting exercises or jumps [5,6,7]. Some authors reported relevant correlations between the PRFD measured in isometric muscle contractions and the same parameter measured in dynamic muscle actions [8,9]. These studies showed that relationships exist between the capacity to generate force rapidly at the isometric midthigh pull (IMTP) and the ability to perform in countermovement jump (CMJ). Not consistently, other authors did not find significant correlations between isometric and dynamic PRFD [10]. These authors found a significant correlation only between sprint performance and peak force during concentric jump squats, but not with other variables of the isometric test. Discrepancies in these results may be partially ascribed to different muscle actions and joint angles between the IMTP and the CMJ [10,11,12]. These differences include neuromuscular activation, force-versus-time relationships, and a role for stretch-shortening cycles. The IMTP primarily evaluates maximum voluntary force and rate of force development (RFD) without changes in joint angles, allowing a direct assessment of neural drive and muscle–tendon unit stiffness [3,13]. In contrast, countermovement jumps (CMJs), with or without external loads or deloads, include complex interactions between eccentric and concentric phases and stretch-shortening cycles that may contribute to differences in force output and RFD in isometric and dynamic tasks. External loads and support mechanisms indeed alter the force–velocity relationship during CMJs, affecting muscle excitation and the characteristics of force development. Additionally, when external loads were applied to the CMJ using weighted vests (CMJ_V_) or elastic bands [14], increases in PRFD and reductions in jump height were registered [8,9]. Consistently, lower PRFD was registered when the CMJ was assisted by elastic bands [14,15]. Despite relevant increases in force production being reported in CMJ_V_ compared to regular CMJ, no increases in power were registered [16]. Recently the isometric mid-shin pull test (MSP) has been proposed as a valid and reliable alternative to the IMTP [13]. The MSP consists of an isometric pull performed by the weightlifting regular barbell height (22.5 cm from the floor to the center of the bar). Large correlations were detected between peak force at MSP and both 1 repetition maximum (1-RM) deadlift and CMJ performance [17]. Moreover, the peak force obtained at MSP was more correlated to CMJ performance compared to the same parameter obtained at IMTP [13]. However, no studies to date have investigated the PRFD and muscle excitation during the regular, band-assisted, band-resisted, and weighted vest CMJs, in comparison to the MSP.

Therefore, the aim of this study was to compare the characteristics of force development and electromyographic activity of the quadriceps muscles between the MSP and the aforementioned CMJs. The authors hypothesized that the PRFD calculated in CMJ may not be higher and not correlated with the same parameter assessed at the MSP test.

## 2. Materials and Methods

### 2.1. Experimental Approach to the Problem

The study protocol consisted of two visits at the Human Performance Laboratory divided by at least 72 h. During the first visit, the participants were familiarized with the assessments included in the study. In the second visit, the participants performed the MSP and the CMJs in four different conditions: regular CMJ, weighted vest CMJ (CMJ_V_), resisted bands CMJ (CMJ_RB_), and assisted bands CMJ (CMJ_AB_). Participants were also asked to abstain from physical activity in the 48 h before the second visit. MSP test and CMJs were performed in randomized order (using https://www.randomizer.org/ accessed on 1 March 2023). Two trials were performed in each exercise and a rest time of 3 min was observed between each trial. Information on muscle excitation during isometric and ballistic actions was obtained by surface electromyography.

### 2.2. Participants

An a priori power analysis was performed using G*Power 3.1.9.7 to determine the required sample size. Parameters were set as follows: effect size f = 0.3 (large), *α* = 0.05, power (1 − β) = 0.8, correlation between repeated measures = 0.5, and no sphericity correction *ε* = 1 [16]. Fifteen experienced resistance-trained individuals (12 men and 3 women) participated in the present study (age = 25.9 ± 4.0 y; body mass = 73.2 ± 11.7 kg; stature = 172.3 ± 9.5 cm; experience = 5.1 ± 4.8 y). Each participant was asked to perform all the assessments included in the protocol. Participants were recruited among gym goers and students at the University courses of Sport Sciences. Inclusion criteria required participants to be between 18 and 35 years old and to be resistance-trained at least three times per week in the two years before the study. Participants reporting injuries that occurred in the 12 months before the study were discarded. The participants signed an informed consent document after being informed of the potential risks of the study. The study was approved by the local Bioethics Committee (n. 0025317; 1 February 2023).

### 2.3. Strength and Power Assessments

Testing sessions were preceded by a standardized warm-up consisting of 5 min of jogging, 10 bodyweight squats, 10 bodyweight walking lunges, 10 dynamic walking hamstring stretches, 10 dynamic walking quadriceps stretches, and 10 bodyweight push-ups [18]. The MSP test was performed on a power rack that permitted fixation of the bar at a height that corresponded to 22.5 cm from the two force plates where the participant was standing (K-Deltas, Kinvent Physio, Montpellier, France, sample frequency: 1000 Hz). The athletes were allowed to choose the most comfortable starting position and were asked to pull as explosively as possible during the MSP test [13]. The participants used lifting straps to improve the bar grip, and the isometric contraction was maintained for 6 **s**. During the isometric measurements, participants were strongly encouraged by the investigators. The force plates were zeroed after every trial and the force–time variables included the individual body mass. Peak force (PF) and peak rate of force development (PRFD) were calculated as the steepest 20 ms portion of the curve [19].

Intraclass coefficients were 0.99 (SEM = 32.56 N) and 0.83 (SEM = 878.5 N s−1) for PF and PRFD at MSP, respectively. The CMJs were performed on the same aforementioned force plates. During the CMJ tests, participants were asked to keep their hands on their hips and to use a self-selected depth to optimize the peak power in their movement [20].

**Figure 1:**
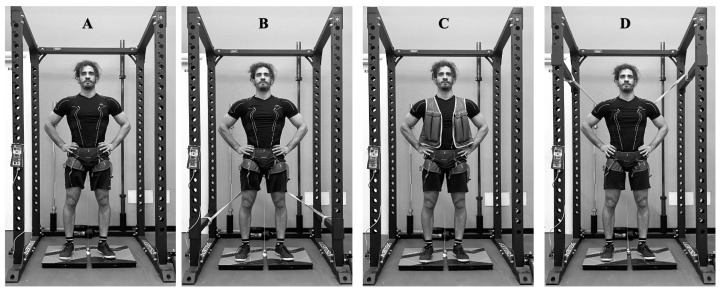
Jumping conditions: in panel (**A**) the CMJ = Countermovement Jump; in panel (**B**) the CMJ_RB_ = resisted band countermovement jump; in panel (**C**) the CMJ_V_ = weighted vest countermovement jump; in panel (**D**) the CMJ_AB_ = assisted band countermovement jump.

As previously described by Makaruk and colleagues [16], the CMJ_V_ was performed with an extra load (weighted vest) corresponding to 10% of the individual body mass. In the CMJ_RB_, resistance bands were attached to the participant to obtain an extra load corresponding to 10% of the individual body mass. Resistance bands were applied to the participants using a climbing harness, as previously described by Argus [14]. The CMJ_AB_ (band-assisted CMJ) was performed with the elastic bands adjusted to reduce individual body mass by 10%. The proper tension of the resistance bands in both CMJ_RB_ and CMJ_AB_ was obtained by adjusting the length of the bands when the participant was standing on the force plate. Each jump condition can be observed in Figure 1.

According to Haff and colleagues [19], in CMJs both PF and PRFD were measured from the ground reaction force data collected by the force plates. Specifically, PRFD was calculated as previously described for the MSP. In the CMJs, a linear encoder (Tendo Unit model V104, Tendo Sports Machines, Trencin, Slovak Republic) was used to measure peak velocity (PV) during the jumps. The best attempt was used to calculate the jump Peak Power (PP) using the following equation [19]:

PP = PV × Peak force including participant’s body mass

The intraclass coefficient calculated for the CMJ Peak Power was 0.96 (SEM = 100.3 W).

### 2.4. Electromyographic Assessments

The skin area under the EMG electrodes was shaved, cleaned, and prepared with conductive cream. After careful preparation of the skin, two pairs of surface electrodes (Ag/AgCl, 30 × 24 mm; H124SG, Cardinal Health, Kendall, UK) were placed at the vastus medialis (EMG_VM_), and vastus lateralis (EMG_VL_) following the European SENIAM guidelines. For the vastus medialis, electrodes were placed at 80% on the line between the anterior iliac spine and the joint space in front of the anterior border of the medial ligament, while for the vastus lateralis, electrodes were positioned at 2/3 on the line from the anterior iliac spine to the lateral side of the patella [21]. A Bluetooth Low Energy 5.1 surface electromyography (K-Myo, Kinvent Physio, Montpellier, France) with a sampling frequency of 1000 Hz was used to detect the muscle signals [22]. The EMG was synchronized with the force plate signals, and both were transmitted to the mobile app Kinvent Physio (version 2.2.1.). To denoise raw EMG data, we applied a 40–400 Hz band-pass Butterworth filter to remove low- and high-frequency noise, and a band-stop filter at 50, 150, and 200 Hz to avoid power line interference and noise from electronic components [23]. EMG signals were normalized using the peak value recorded during each session to attribute amplitude variations to muscle activity rather than extrinsic factors. The root mean square (RMS) was calculated in a 25-millisecond mobile window [24] for each repetition. The average muscle excitation of the 6 s MSP test and the muscle detected during the concentric phase of the CMJs were used for subsequent analysis.

### 2.5. Statistical Analysis

A Shapiro–Wilk test was used to test the normal distribution of the data. The differences in strength and EMG parameters between the testing conditions were calculated using a one-way analysis of variance (ANOVA) with repeated measures. If the assumption of sphericity was violated, a Greenhouse Geisser correction was applied. Pairwise comparisons were performed using Bonferroni’s post hoc. A preliminary correlation analysis was performed using Pearson’s correlation. The magnitude of the correlations was evaluated as trivial (0–0.09), small (0.1–0.29), moderate (0.3–0.49), large (0.5– 0.69), very large (0.7–0.89), near perfect (0.9–0.99), and perfect (=1) [25]. In post hoc analysis Cohen’s d effect sizes (ES) were calculated to determine the magnitude of differences: small > 0.2, medium > 0.6, large > 1.20, very large > 2 [25,26]. A significant level of *p* ≤ 0.05 was used and all data were reported as mean ± SD. Data were analyzed using SPSS Software v. 28.0 for Windows (SPSS Inc., Chicago, IL, USA).

## 3. Results

**Figure 2:**
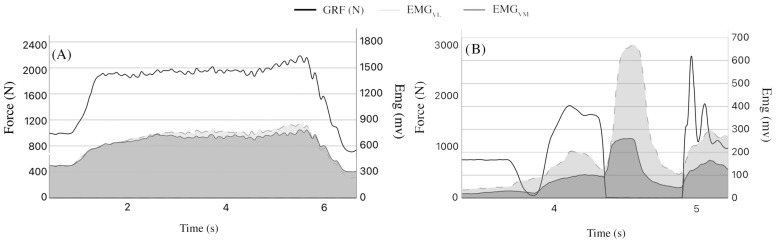
Examples of time–force curve and EMG signals from vastus medialis and vastus lateralis during MSP in panel (**A**), and during CMJs in panel (**B**).

Figure 2 shows a typical example of synchronization of the EMG recordings of the vastus medialis and vastus lateralis during CMJ and MSP. All the data related to performance assessments were normally distributed (*p* < 0.05). Results of performance assessments and muscle excitation are reported in Table 1.

### 3.1. Strength and Power Assessments

**Figure 3:**
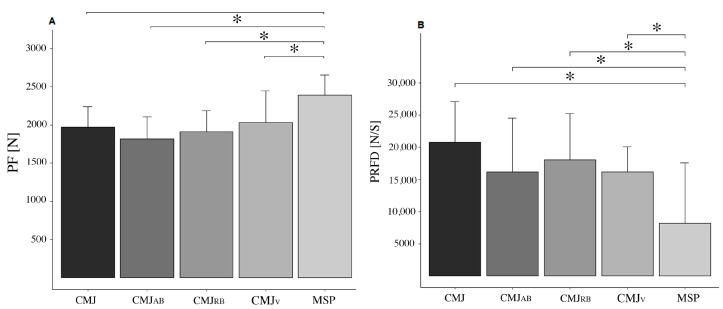
Panel (**A**) shows the Peak Forces (PF), and panel (**B**) shows the Peak Rate of Force Development (PRFD) produced in the regular countermovement jump (CMJ); CMJ_AB_ = assisted band CMJ; CMJ_RB_ = resisted bands CMJ; CMJ_V_ = weighted vest CMJ, and during the mid-shin pull (MSP) test. All data are reported as mean and error bars represent SD. * Indicates a significant difference (*p* < 0.05) between conditions.

**Figure 4:**
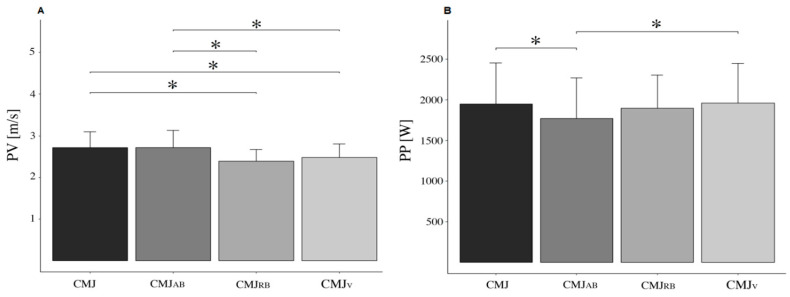
Panel (**A**) shows the Peak Velocity (PV), and panel (**B**) shows the Peak Power (PP) produced in the regular countermovement jump (CMJ), in CMJ_AB_ = assisted band CMJ, in CMJ_RB_ = resisted band CMJ, and in CMJ_V_ = weighted vest CMJ. All data are reported as mean and error bars represent SD. * Indicates a significant difference (*p* < 0.05) between the conditions.

A significant difference in PF was detected between the assessments performed (*p* < 0.001; F = 20.055; η^2^ = 0.589). Post hoc analysis showed significant differences between MSP and CMJ (*p* < 0.001; ES = 1.384), CMJ_AB_ (*p* < 0.001; ES = 1.836), CMJ_RB_ (*p* < 0.001; ES = 1.567), and CMJ_V_ (*p* < 0.001; ES = 1.203). The lowest values of PF were detected in the jumps (without significant differences between them), while the highest values were registered in the MSP. A significant difference was also detected for the PRFD (*p* < 0.001; F = 6.709; η^2^ = 0.324). Post hoc analysis indicated a significant difference in PRFD between the MSP test and the CMJ (*p* = 0.001; ES = −1.722), CMJ_AB_ (*p* = 0.029; ES = −1.091), CMJ_RB_ (*p* = 0.003; ES = −1.351), and CMJ_V_ (*p* = 0.030; ES = −1.089). No significant differences in PRFD were detected between the jump conditions (*p* > 0.05). Results for PF and PRFD can be observed in Figure 3. Differences in the PV were detected between the CMJ conditions (*p* < 0.001; F = 13.20; η^2^ = 0.545). In particular, PV was more elevated in CMJ compared to CMJ_RB_ (*p* < 0.001; ES = 0.93), in CMJ compared to CMJ_V_ (*p* = 0.006; ES = 0.658), in CMJ_AB_ compared to CMJ_RB_ (*p* < 0.001; ES = 0.917), and in CMJ_AB_ compared to CMJ_V_ (*p* = 0.007; ES = 0.644). The PP also differed in the CMJs (*p* < 0.001; F = 7.099; η^2^ = 0.392), with a significant difference detected between CMJ and CMJ_AB_ (*p* = 0.003; ES = 0.376) and between CMJ_AB_ and CMJ_V_ (*p* = 0.002; ES = −0.394). The results for PV and PP measured in the different CMJs are reported in Figure 4.

### 3.2. Electromyographic Assessments

**Figure 5:**
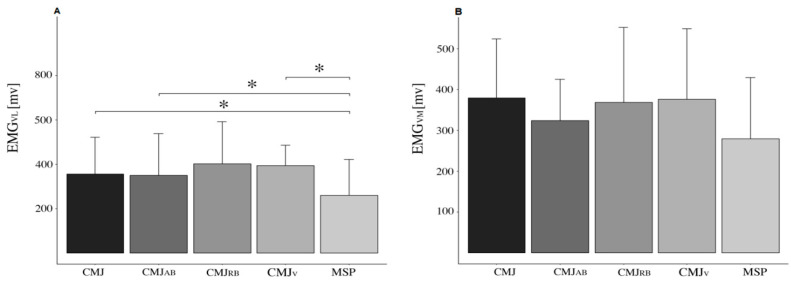
Panel (**A**) shows EMG_VL_ = muscle excitation of Vastus Lateralis, and panel (**B**) the EMG_VM_ = muscle excitation of Vastus Medialis, during the regular countermovement jump (CMJ), during the CMJ_V_ = weighted vest CMJ, during the CMJ_RB_ = resisted band CMJ, and during the CMJ_AB_ = assisted band CMJ, and during the mid-shin pull (MSP) test. All data are reported as means, and error bars represent SD. * Indicates a significant difference (*p* < 0.05) between the conditions.

Differences between the conditions were detected for EMG_VL_ (*p* < 0.001; F = 6.624; η^2^ = 0.321). Pairwise comparisons indicated that muscle excitation was more elevated in CMJ (*p* = 0.030; ES = 0.593), CMJ_RB_ (*p* < 0.001; ES = 0.875), and CMJ_V_ (*p* < 0.001; ES = 0.826) compared to MSP. No significant differences in EMG_VM_ were detected between the conditions (*p* = 0.087; F = 2.714; η^2^ = 0.162). All EMG results are reported in Figure 5.

### 3.3. Correlations Between Variables

No significant correlations were found between MSP and CMJs for the PRFD (*p* > 0.05). However, a significant correlation was found in the PRFD measured at CMJ_AB_ and CMJ_RB_ (r = 0.601; *p* = 0.018). In addition, large and very large correlations were detected between the PRFD registered in MSP and the PV measured in CMJ_AB_ (r = 0.786; *p* = 0.002), and CMJV (r = 0.676; *p* = 0.016). Large correlations were also found between the PRFD at the MSP and the PP registered in the CMJ (r = 0.757; *p* = 0.004), CMJ_AB_ (r = 0.784; *p* = 0.003), CMJ_RB_ (r = 0.685; *p* = 0.014), and CMJ_V_ (r = 0.833; *p* < 0.001). Correlations between PF at MSP and PP in CMJ (r = 0.658; *p* = 0.020), in CMJ_AB_ (r = 0.608; *p* = 0.036), in CMJ_RB_ (r = 0.620; *p* = 0.031), and CMJ_V_ (r = 0.732; *p* = 0.007) were found. No correlations were found between PF at MSP and PV in CMJs (*p* > 0.05). Additionally, the PRFD at MSP was significantly correlated with EMG_VL_ at CMJ (r = 0.534; *p* = 0.040), at CMJ_RB_ (r = 0.593; *p* = 0.020), and at CMJ_V_ (r = 0.762; *p* < 0.001). No other significant correlations between the variables were detected.

## 4. Discussion

The aim of the present study was to investigate the characteristics of force production and muscle activation in the CMJ test performed at different loads in comparison with the isometric mid-shin pull test (MSP). Results showed lower peak forces (PF) registered at the beginning of the pushing phase of the CMJ compared to the isometric PF detected at the MSP test. In MSP and CMJ the vertical component of the ground reaction force, which was recorded as PF, is the result of different mechanisms. While in MSP, the 6 s isometric contraction of the upper and lower body muscles and the individual body mass were converted into the registered values of PF; in the CMJ, the acceleration of the body mass was responsible for the increase in PF during the jump. Another difference between the force produced in MSP and in CMJs is represented by the duration of the muscle contraction. In CMJ, indeed, the ability to develop force rapidly represents a key component of performance [27], while this factor is not crucial in the MSP. This discrepancy in the time of muscle contraction may partially explain the higher values of force registered in MSP compared to CMJs.

The crucial role of rapid force production in CMJ is confirmed by the higher values of PRFD detected in CMJs compared to MSP. Although the participants were asked to pull the bar as fast as they could and were familiar with the MSP assessment, they were not able to obtain similar values of PRFD compared to CMJs. Despite the difficulty of performing a real maximum explosive contraction at the MSP, our results confirm other authors [19] who studied PRFD in dynamic and static muscle actions. In addition, the PRFD measured in MSP was not correlated with the same parameter registered in any of the CMJs performed. This is also consistent with other studies [17], which suggest that different mechanisms of motor unit recruitment may exist in isometric and dynamic muscle actions. According to these authors, a large correlation was detected between the PF registered at MSP and the PP measured in the CMJs (r = 0.608–0.732). Resistance bands (assisting or resisting the CMJ) and loaded vests were not able to significantly influence the PRFD registered during the jumps. On the contrary, PV was significantly lower when the CMJ was resisted by bands or when the participants wore a weighted vest. Resistance obtained by elastic bands increases with the length of the band and is typically defined as variable [28]. On the contrary, additional loads provide a constant external resistance in the entire range of motion and respond to the principle of inertia. Despite the difference between the resistance produced by additional load (CMJ_V_) and elastic bands (CMJ_RB_), no differences were detected between these conditions in the PRFD, PP, and PV. When the load was reduced by elastic bands assisting the CMJ (CMJ_AB_), PV was the same as CMJ, but power was reduced. Power indeed represents a combination of load and velocity [29,30] and external load was lower in CMJ_AB_ compared to the regular CMJ. These results are consistent with Fernandes et al. [15], who found similar decreases in power when elastic bands were assisting the jump. In the present study, a reduction of 10% of the individual body mass was obtained in CMJ_AB_ by using resistance bands. Tran et al. [31], however, suggested a range of body mass reduction between 10% and 30% of the participant's body mass to induce significant changes in jumping variables. Thus, a reduction by 10% of the body mass may not be enough to alter the jumping force–time characteristics in resistance-trained individuals. This is also confirmed by the absence of differences between muscle excitation of VM and VL between the different CMJs. However, in both regular, assisted, or resisted CMJ, the excitation of VM was significantly higher compared to MSP. Lower muscle excitations registered in MSP compared to CMJ, may be related to different hip and knee angles influencing the activation of the gluteus muscles. The deep squat position and the trunk inclination that characterize the MSP may indeed activate the gluteus to a greater extent than the leg extensor muscles [32]. Thus, the lack of assessment of the excitation of the hip extensor muscles and the absence of information about hip and knee joint angles during both isometric and dynamic assessments represent possible limitations of the present study.

## 5. Conclusions

In conclusion, the present investigation provided further evidence that the CMJs and MSP tests assess distinct aspects of neuromuscular performance. This aspect is of crucial importance when selecting the most appropriate test to assess a particular aspect of an athlete’s power and strength performance. While MSP elicited higher peak force due to its isometric nature and longer contraction times, the CMJ highlighted the importance of rapid force development and velocity. Assisted or resisted CMJs did not significantly enhance PRFD or PF compared to regular CMJ. These findings emphasize the utility of MSP for developing maximum strength and CMJ for explosive strength and power in trained individuals. Further research is necessary to explore these variables and enhance our understanding of dynamic and isometric performance.

## 6. Practical Applications

Results of the present study showed that assisting the CMJ with resistance bands may promote peak velocity but does not promote power development and PRFD. As previously reported by Fernandes and colleagues [15], the regular CMJ performed at body mass may represent the best option for power development in trained individuals. Resisted band CMJ and the use of weighted vests to increase the external load in the CMJ by 10% of body mass, do not seem to significantly increase peak force and muscle excitation of knee extensors compared to regular CMJ. On the contrary, the MSP is characterized by higher force production and longer time under tension compared to the CMJ. However, lower PRFD and muscle excitations of the knee extensors are typically detected in MSP compared to the CMJ. Thus, isometric, whole-body exercises, such as the MSP, may be effective in developing maximum strength but less appropriate for stimulating explosive strength parameters, such as the PRFD.

## Figures and Tables

**Table 1:** Parameters of performance and muscle excitation acquired in the different assessments. CMJ = Countermovement Jump; CMJ_AB_ = assisted band countermovement jump; CMJ_RB_ = resisted band countermovement jump; CMJ_V_ = weighted vest countermovement jump; MSP = Mid-shin Pull; PF = Peak Force; PRFD = Peak Rate of Force Development; PV = Peak Velocity; PP = Peak Power; EMG_VM_ = muscle excitation of Vastus Medialis; EMG_VL_ = muscle excitation of Vastus Lateralis; * indicates a significant difference with MSP (*p* < 0.05).

	CMJ	CMJ_AB_	CMJ_RB_	CMJ_V_	MSP
PF (N)	1968.73 ± 263.67 *	1819.4 ± 270.00 *	1908.37 ± 288.47 *	2028.57 ± 278.17 *	2426.29 ± 493.402
PRFD (N*s^−1^)	20,739.43 ± 9451.06 *	16,120.5 ± 6328.96 *	18,026.26 ± 8409.46 *	16,109.06 ± 7207.9 *	8136.25 ± 3968.85
PV (m*s^−1^)	2.71 ± 0.38	2.71 ± 0.41	2.38 ± 0.29	2.48 ± 0.33	
PP (W)	1949.03 ± 501.26	1770.2 ± 495.42	1895.78 ± 408.15	1957.63 ± 490.51	
EMG_VM_ (mV)	379.66 ± 143.45	323.3 ± 101.7	368.63 ± 183.03	376.08 ± 172.3	279.45 ± 150.17
EMG_VL_ (mV)	357.02 ± 163.04 *	351.18 ± 166.14 *	403.12 ± 187.39	395.13 ± 189.39 *	260.17 ± 91.57

## Data Availability

The raw data supporting the conclusions of this article will be made available by the authors on request.

## Abbreviations

**Abbreviations**	**Full Name**
MSP	Midshin pull
CMJ	Countermovement jump
CMJ_RB_	Countermovement jump with resisted bands
CMJ_AB_	Countermovement jump with assisted bands
CMJ_V_	Countermovement jump with weighted vest
PF	Peak force
PV	Peak Velocity
PP	Peak Power
PRFD	Peak Rate of Force Development
EMG_VL_	Electromyography of Vastus Lateralis
EMG_VM_	Electromyography of Vastus Medialis
